# Ornipural^®^ Mitigates Malathion-Induced Hepato-Renal Damage in Rats via Amelioration of Oxidative Stress Biomarkers, Restoration of Antioxidant Activity, and Attenuation of Inflammatory Response

**DOI:** 10.3390/antiox11040757

**Published:** 2022-04-11

**Authors:** Osama S. El Okle, Hossam G. Tohamy, Saed A. Althobaiti, Mohamed Mohamed Soliman, Heba I. Ghamry, Foad Farrag, Mustafa Shukry

**Affiliations:** 1 Departement of Forensic Medicine and Toxicology, Faculty of Veterinary Medicine, Alexandria University, Alexandria 22758, Egypt; osama.forensics@alexu.edu.eg; 2 Departement of Pathology, Faculty of Veterinary Medicine, Alexandria University, Alexandria 22758, Egypt; hossam.gafaar@alexu.edu.eg; 3 Biology Department, Turabah University College, Taif University, Taif 21995, Saudi Arabia; saed@tu.edu.sa; 4 Clinical Laboratory Sciences Department, Turabah University College, Taif University, Taif 21995, Saudi Arabia; mmsoliman@tu.edu.sa; 5 Department of Home Economics, College of Home Economics, King Khalid University, P.O. Box 960, Abha 61421, Saudi Arabia; hgmry@kku.edu.sa; 6 Department of Anatomy and Embryology, Faculty of Veterinary Medicine, Kafrelsheikh University, Kafrelsheikh 33516, Egypt; foad.farrag@vet.kfs.edu.eg; 7 Department of Physiology, Faculty of Veterinary Medicine, Kafrelsheikh University, Kafrelsheikh 33516, Egypt

**Keywords:** malathion, hepato-renal damage, Ornipural^®^, metabolic parameters, antioxidant, inflammation

## Abstract

The current study was instigated by investigating the ameliorative potential of Ornipural^®^ solution against the hepato-renal toxicity of malathion. A total number of 35 male Wistar albino rats were divided equally into five groups. Group 1 served as control and received normal saline intraperitoneally. Group 2, the sham group, were administered only corn oil (vehicle of malathion) orally. Group 3 was orally intoxicated by malathion in corn oil at a dose of 135 mg/kg BW via intra-gastric gavage. Group 4 received malathion orally concomitantly with Ornipural^®^ intraperitoneally. Group 5 was given Ornipural^®^ solution in saline via intraperitoneal injection at a dose of (1 mL/kg BW). Animals received the treatment regime for 30 days. Histopathological examination revealed the harmful effect of malathion on hepatic and renal tissue. The results showed that malathion induced a significant decrease in body weight and marked elevation in the activity of liver enzymes, LDH, and ACP. In contrast, the activity of AchE and Paraoxonase was markedly decreased. Moreover, there was a significant increase in the serum content of bilirubin, cholesterol, and kidney injury markers. A significant elevation in malondialdehyde, nitric oxide (nitrite), and 8-hydroxy-2-deoxyguanosine was observed, along with a substantial reduction in antioxidant activity. Furthermore, malathion increased tumor necrosis factor-alpha, the upregulation of IL-1B, BAX, and IFN-β genes, and the downregulation of Nrf2, Bcl2, and HO-1 genes. Concurrent administration of Ornipural^®^ with malathion attenuated the detrimental impact of malathion through ameliorating metabolic biomarkers, restoring antioxidant activity, reducing the inflammatory response, and improving pathologic microscopic alterations. It could be concluded that Ornipural^®^ solution demonstrates hepatorenal defensive impacts against malathion toxicity at biochemical, antioxidants, molecular, and cellular levels.

## 1. Introduction

Malathion is one of the earliest organophosphate insecticides developed globally and is still extensively used in Egypt, mainly for agricultural purposes. Acute toxicity of malathion is particularly related to the nervous system. It is characterized by the inactivation of acetylcholinesterase (AChE) and butyrylcholinesterase enzymes, which result in overstimulation of the cholinergic pathways [1]. Even in small doses, prolonged exposure to malathion is usually associated with pronounced hepatic and renal disorders in lab animals [2,3]. Malathion caused acute oxidative stress in rat brains at doses as low as 25 mg/kg [4]. Malathion caused chronic oxidative damage in the brain (5 and 10 mg/kg daily for 28 days) [5]. Malathion also caused neurobehavioral abnormalities and neuronal death following subacute dermal exposure (44 mg/kg, for 30 days) [6].

For a long time, it was established that the antidotal treatment of acute malathion toxicity consists of administering anticholinergic drugs, such as atropine, in combination with AChE reactivator (oximes), such as pralidoxime [7]. However, there was a limited investigatory trial conducted to treat or prevent malathion-induced hepatic and renal damage. Therefore, the primary novel objective of the present study is to investigate the protective efficacy of commercial veterinary product Ornipural^®^ against malathion-induced hepato-renal toxicity in rats.

Widespread use of malathion leads to environmental pollution, and increases the extent of exposure [8,9]. Malathion exposure can cause acute or chronic toxicity, especially in developing countries [5]. Workers of malathion factories are prone to malathion intoxication [10].

Since malathion is lipophilic, it is rapidly absorbed and distributed to various organs, causing multiple pathologies [11,12]. Malathion-induced oxidative stress has recently been discovered in numerous human cell types [13,14]. It has been found that malathion treatment causes an increase in reactive oxygen species (ROS) production and lipid peroxidation [15]. Malathion is the fourth most effective neurotoxin [15]. Endogenous enzymatic and nonenzymatic antioxidant activities in brain tissues are altered by malathion [16]. Malathion-induced oxidative stress can cause mitochondrial dysfunction, DNA breaks, autophagy, and apoptosis by increasing oxidative stress markers [8]. Malathion induces hepatocellular injury in liver tissue and upsurged liver enzymes [17]. Malathion exposure may be related to a higher chance of developing atherosclerosis [18].

Hepatic glycogenolysis and gluconeogenesis may be activated by malathion, resulting in hyperglycemia. Hyperglycemia caused by malathion can also be explained in terms of its hepatic toxic inflammatory effects [19], as well as by increases renal injury markers [2], leading to toxicity in endocrine disruptors [20,21]. Malathion has been shown to induce anemia [19]. Malathion is a recognized mutagenic agent that targets DNA [22]. 

The WHO categorizes malathion as a slightly hazardous insecticide [20], and endorses a dosage of 2 g/m^2^, giving a residual effect of 60–90 days, and the WHO/FAO’s maximum daily intake standard (0.02 mg/kg/day) [21]. The Environmental Protection Agency (EPA) estimates that malathion is used for more than 30 million pounds of crops per year. It is s used on various food crops, including cotton and rice. It’s used on different crops, including cotton and rice [22].

The pharmacological properties of Ornipural^®^ that are commonly used in veterinary fields are activators for liver function, lipotropic medication, and diuretic medication. Ornipural^®^ is a unique commercial formulation that contains many substances that possess hepatic and renal protective properties: betaine (15 mg), arginine (hydrochloride) (33.3 mg), ornithine (hydrochloride) (11.8 mg), citrulline (10 mg), sorbitol (200 mg), and metacresol (3 mg). Betaine is a lipotropic factor, participating in the fight against fatty overload and hepatic steatosis of the liver [23]. Sorbitol is a carbohydrate that improves the intestinal absorption of specific vitamins, particularly B12 and B6 and ferric ions. It is also a nutritional contribution and a diuretic [24]. Ornithine and citrulline are amino acids used as detoxification factors in the body by activating the ureagenesis cycle [25]. Arginine, another amino acid, is part of the Krebs cycle, and facilitates ureagenesis [26]. Therefore, the present study was constructed to investigate the protective efficacy of Ornipural^®^ against malathion-induced hepatorenal injury biochemical markers, gene expression of oxidative and inflammatory mediators, and apoptosis, in addition to histopathology in rats.

## 2. Materials and Methods

### 2.1. Chemicals

O-dimethyl phosphorodithioate of diethyl mercaptosuccinate, commercially available as malathion (98% active ingredient), was obtained from Kafr El Zayat, Egypt. Ornipural^®^ solution (Vetoquinol, Magny-Vernois, France) was purchased commercially from a local veterinary clinic (Sakkah, Kafrelsheikh, Egypt).

### 2.2. Experimental Animals, Treatment Protocol, and Ethical Considerations

The present experiment was performed on 35 male Wistar albino rats weighing (147 ± 5 g, six weeks of age). Animals were obtained from the Medical Research Institute, Alexandria University, Egypt, and were provided with a standard diet and water ad libitum. They were maintained under supervision, particularly in metallic rat cages with 12 h light-dark cycle at 27 °C ± 2 °C for one week as an acclimatization before the treatment. After the acclimatization period, rats were randomly and equally divided into five experimental groups (*n* = 7). Group 1 was kept as a control, and received normal saline intraperitoneally. Group 2, the sham group, was administered only standard corn oil (vehicle of malathion) orally. Group 3 was orally intoxicated by malathion in corn oil at 135 mg/kg (1/10 of the oral LD50 in male rats) [27] via intra-gastric gavage according to the manufacturing guide; group 4 received both malathion orally and Ornipural^®^ intraperitoneally following the manufacturing guide. Group 5 was given Ornipural^®^ solution by intraperitoneal injection at a dose of (1 mL/kg BW).

Treatments were given daily and prolonged for 30 days. On the last day of the experiment, the final body weight of rats was measured using a digital scale; then, animals were anesthetized with a combination of ketamine and xylazine injection. Blood was collected from the orbital venous plexus, then was centrifuged at 3000 rpm for 10 min to separate serum, and stored at −20 °C for hepatic and renal functional biomarkers analysis. Rats were sacrificed by decapitation, then livers and kidneys were collected, weighed, and washed in ice-cold saline. A small piece of both tissues was fixed in a 10% neutral-buffered formalin solution for histopathological examination. Another piece from the livers and kidneys was stored at −20 °C until the assessment of oxidative stress markers, while the last part was stored in liquid nitrogen at −80 °C and subjected to gene expression evaluation. Experimental procedures were approved by Alexandria university’s Institutional Animal Care and Use Committee (ALEXU-IACUC, 09082021), and followed ethical guidelines of the Faculty of Veterinary Medicine, Alexandria University.

### 2.3. Assessment of Hepato-Renal Functional and Metabolic Serum Parameters

The activity of the enzymes aspartate aminotransferase (AST) and alanine aminotransferase (ALT) in the serum was measured following [28]; the alkaline phosphatase (ALP) was determined using the method of [29]. Total protein and albumin levels were measured [30]. After subtracting albumin from the total protein, serum globulin was estimated to indicate liver damage. Serum creatinine and urea levels were also tested as kidney function indicators [31,32]. Serum uric acid was assessed following [33].

Acid phosphatase (ACP) was analyzed using Diamond Diagnostics kits, Egypt. Lactate dehydrogenase (LDH) was determined in serum samples using ELISA kits (Wuhan EIAab Science Co. (Wuhan, China); Catalogue No; E1864r). Using commercially available kits, serum uric acid, creatinine, and urea were spectrophotometrically measured (Spinreact, S.A., Girona, Spain).

Boehringer Mannheim colorimetric kits were used to measure serum triglycerides (TG) and total cholesterol (TC) (Mannheim, Germany). HDL-C was also evaluated following the Lopes-Virella et al. [34] method. One serum aliquot was precipitated with phosphotungstic acid and magnesium chloride; afterwards, the cholesterol content was assessed in the clear supernatant using the Boehringer–Mannheim kit (Mannheim, Germany). After that, LDL-C was computed using the Friedewald, et al. [35] equation:

$$\mathrm{LDL}-C=\mathrm{TC}-\left(\mathrm{HDL}-C+\frac{1}{5}\mathrm{TG}.\right)$$

The quantity of acetylcholinesterase (AChE) in supernatants was measured using an ELISA kit purchased from NOVA. (Bioneovan Co., Ltd., DaXing Industry Zone, Beijing, China) following the manufacturer’s instructions. The serum’s paraoxonase (PON) activities were measured using an autoanalyzer and commercially available kits (Rel Assay, Gaziantep, Turkey). (Cobas Integra 800, Roche, Basel, Switzerland). An ammonia assay kit was used to determine the quantity of ammonia (Abcam, Cambridge, UK).

### 2.4. Investigation of Hepato-Renal Oxidant and Antioxidant Tissue Parameters

Quantitative data of pro-inflammatory cytokines such as tumor necrosis factor-alpha (TNF-α; Catalog Number: EZMTNFA, Millipore, Burlington, MA, USA). Lipid peroxidation (LPO) were spectrophotometrically detected in terms of malondialdehyde (MDA) generation following Ohkawa et al. [18]. Nitric oxide (NO) was measured by the colorimetric method of Green et al. [19]. Glutathione (GSH) was estimated following Ellman [20], using the capability of GSH to reduce 5,5′-dithiobis (2-nitrobenzoic acid), forming a yellow compound spectrophotometrically measured at 405 nm. The action of superoxide dismutase (SOD) and catalase (CAT) were estimated to be inconsistent with Sun et al. [21] and Aebi [22], respectively. Glutathione peroxidase (GPx) activity was assayed by the principles of Paglia and Valentine [23].

One of the most significant markers of oxidant-induced DNA damage is 8-hydroxydeoxyguanosine (8-OHdG). The analysis of (8-OHdG) was carried out with the help of OxiSelect™ Oxidative DNA Damage ELISA Kit (Cell Biolabs, San Diego, CA, USA), following the manufacturer’s instructions.

### 2.5. Assay of Pro-Inflammatory Cytokines Gene Expression in Livers and Kidneys

According to the manufacturer’s instructions, total RNA was isolated with the TRIzol reagent (Life Technologies, Gaithersburg, MD, USA). cDNA was immediately prepared using the MultiScribe RT enzyme kit (Applied Biosystems, Foster City, CA, USA). The resulting cDNA was subjected to triplicate real-time PCR analysis. Real-time PCR reactions were performed using Power SYBR Green PCR Master Mix (Applied Biosystems, Life Technologies, CA, USA) on a 7500 Real-Time PCR Systems (Applied Biosystems, Foster City, CA, USA).

The relative fold change in the mRNA expression of examined genes was compared to the control. The expression of β-actin, a standard housekeeping gene, was used to normalize the fold change in mRNA expression of measured genes. Primer sequences and accession numbers of the genes are given in Table 1.

### 2.6. Histopathological Examination

Liver and kidney tissue specimens were processed through the conventional paraffin embedding technique [36]. Sections measuring 5 µm in thickness were obtained from paraffin blocks and stained with hematoxylin and eosin (HE), then examined under a light microscope.

### 2.7. Statistical Analysis

All data were examined by one-way analysis of variance using SPSS (version 25). Data were presented as means ± S.E.M., and *p* values < 0.05 were considered significant. Multiple range comparisons with Duncan’s multiple range test were used to analyze the significant main effects of experimental treatment.

## 3. Results

### 3.1. Performance and Relative Weight of Livers and Kidneys

As shown in Table 2, rats intoxicated by malathion suffered significantly decreased final body weight and weight gain, compared all other groups. The absolute and relative weight of the liver and kidney did not exhibit significant deviation from normal control values. Data also represented that the co-administration of Ornipural^®^ with malathion relatively counteracts the harmful effect of malathion on the growth performance of animals.

### 3.2. Hepato-Renal Functional and Metabolic Serum Parameters

As shown in Table 3, the analysis of serum obtained from animals exposed to malathion showed a significant elevation in ALT, AST, ALP, LDH, and ACP activities. In contrast, the activities of AchE and Paraoxonase were markedly decreased. Moreover, there was a significant increase in total bilirubin, cholesterol, uric acid, urea, creatinine, and ammonia. At the same time, the content of total protein, albumin, and triglycerides was significantly decreased relative to that in the healthy control. In contrast, the tested parameters in the serum of rats given either corn oil or Ornipural^®^ did not deviate significantly from the control data. Obtained data also showed the efficacy of Ornipural^®^ in restoring malathion-induced hepatic, renal, and metabolic biochemical deterioration.

### 3.3. Evaluation of Oxidative Stress in Liver and Kidney Tissue

**Figure 1:**
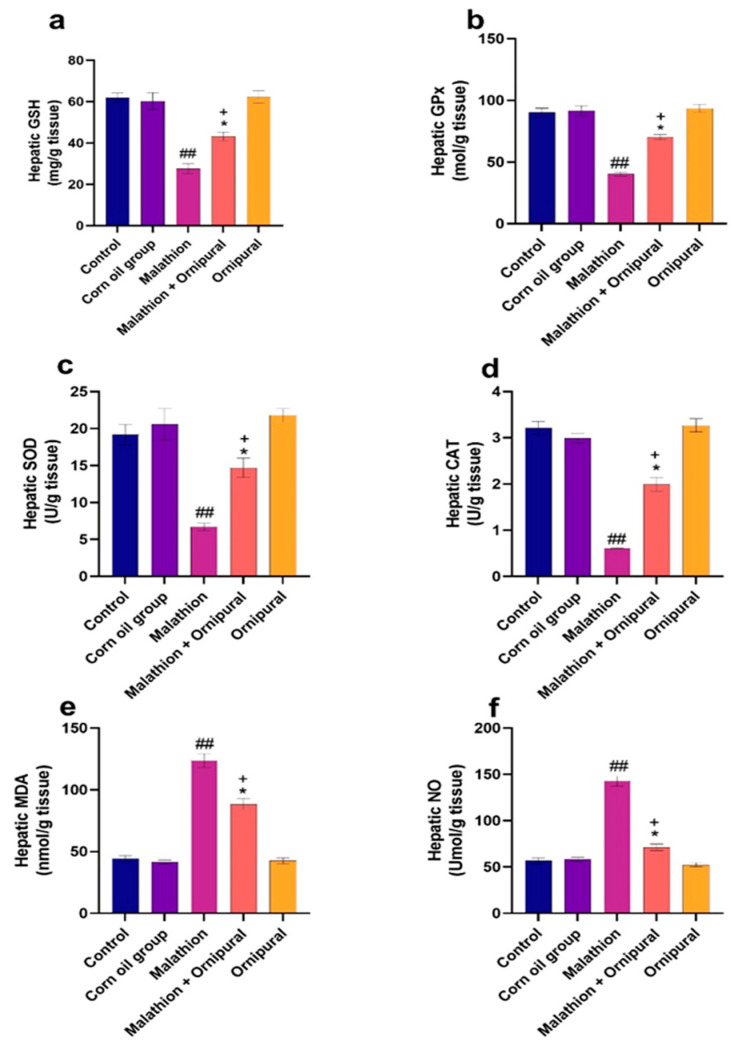
Modulatory effect of Ornipural^®^ against malathion-induced oxidative stress in hepatic tissue (**a**) G.S.H. (**b**) G.P.X. (**c**) S.O.D. (**d**) C.A.T. (**e**) M.D.A. (**f**) NO. The statistical analysis was performed using one-way ANOVA, followed by Duncan’s multiple range test. Data are expressed as mean + S.E.M. Data are expressed as mean + SEM. ^##^ *p* < 0.01 control/sham vs. malathion. * *p* < 0.05 malathion vs. malathion and Ornipural^®^. ^+^ *p* < 0.05 control/sham vs. Malathion and Ornipural^®^.

**Figure 2:**
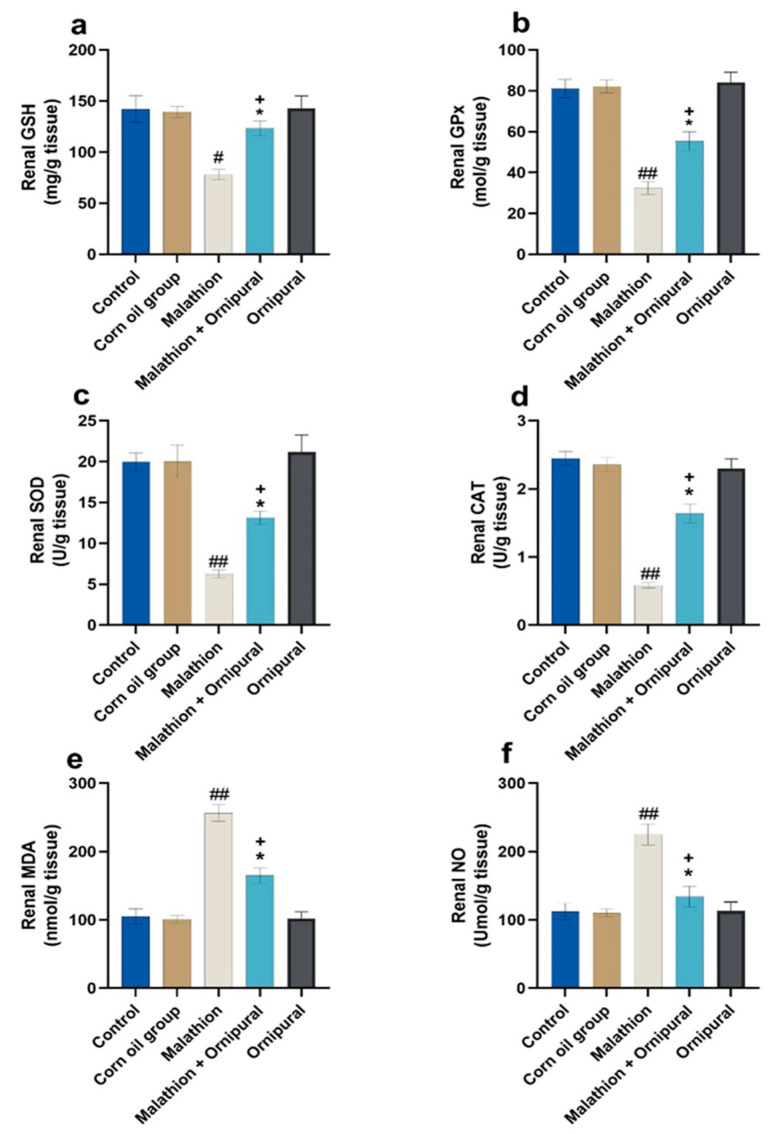
Modulatory effect of Ornipural^®^ against malathion-induced oxidative stress in renal tissue (**a**) G.S.H. (**b**) G.P.X. (**c**) S.O.D. (**d**) C.A.T. (**e**) M.D.A. (**f**) NO. The statistical analysis was performed using one-way ANOVA, followed by Duncan’s Multiple range test. Data are expressed as mean + SEM. ^#^ *p* < 0.05, and ^##^ *p* < 0.01 control/sham vs. malathion. * *p* < 0.05 malathion vs. malathion and Ornipural^®^. ^+^ *p* < 0.05 control/sham vs. malathion and Ornipural^®^.

As illustrated in Figure 1 and Figure 2, malathion intoxication significantly increases lipid peroxidation, MDA, and NO production in liver and kidney tissues. At the same time, the content of GSH and the activity of GPX, SOD, and CAT were significantly decreased relative to other experimental groups. In addition, animals given Ornipural^®^ concurrently with malathion showed marked protection against both the depletion of antioxidant enzymes activity and the elevation of oxidative damage products in hepatic and renal tissues.

### 3.4. Inflammatory Cytokines Genes Expression

**Figure 3:**
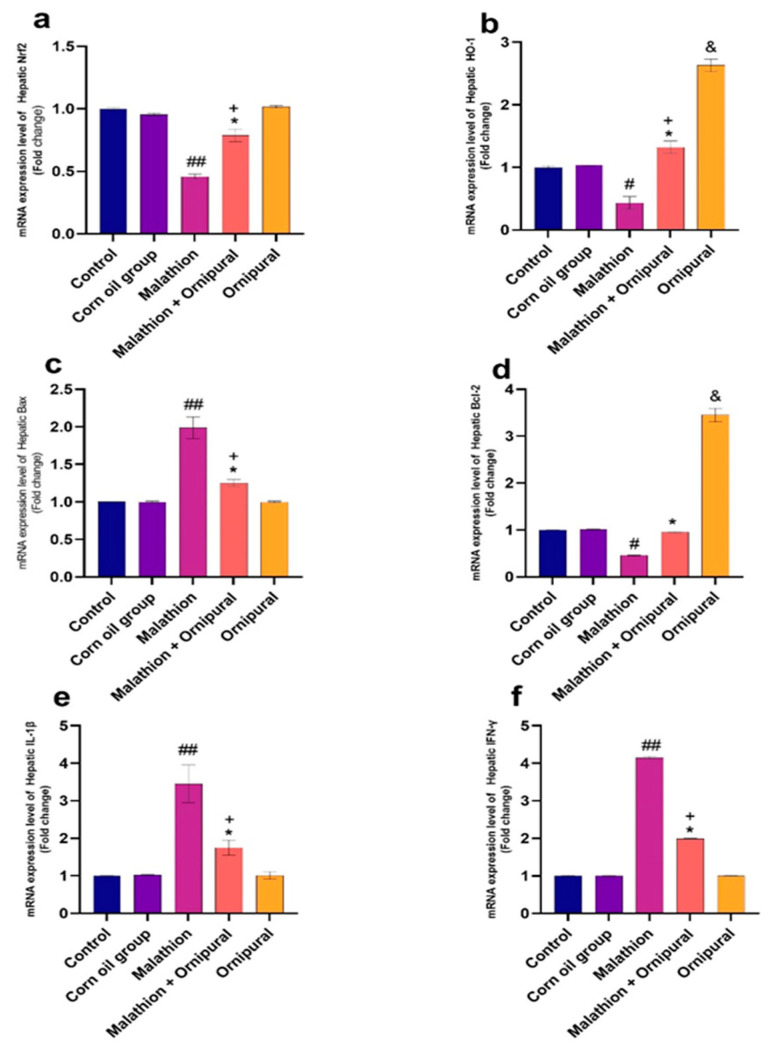
Modulatory effect of Ornipural^®^ against malathion-induced inflammation in hepatic tissue gene expression (**a**) Nrf2 (**b**) HO-1 (**c**) Bax (**d**) Bcl-2 (**e**) IL-1β (**f**) IFN-γ. Data are expressed as mean ± S.E.M. The statistical analysis was performed using one-way ANOVA, followed by Duncan’s multiple range test ^#^ *p* < 0.05, and ^##^ *p* < 0.01 Control/sham vs. malathion. * *p* < 0.05 malathion vs. malathion and Ornipural^®^. ^+^ *p* < 0.05 control/sham vs. malathion and Ornipural^®^. ^&^ *p* < 0.05 control/sham vs. Ornipural^®^.

**Figure 4:**
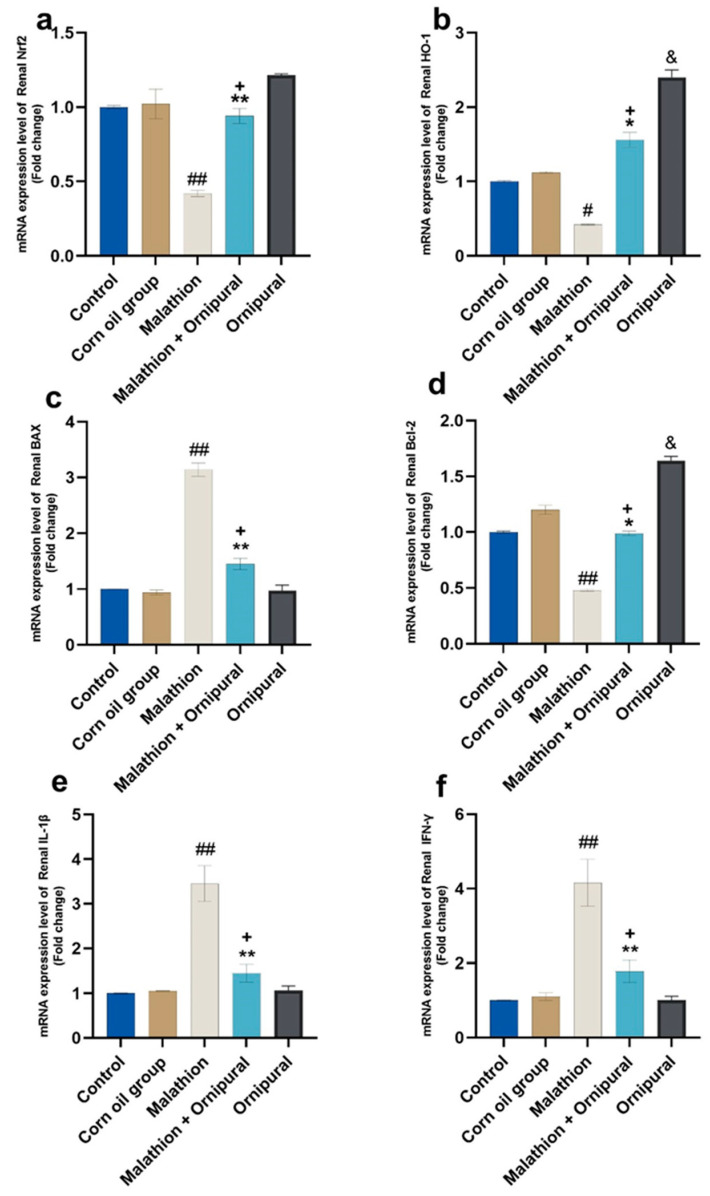
Modulatory effect of Ornipural^®^ against malathion-induced inflammation in renal tissue gene expression (**a**) Nrf2 (**b**) HO-1 (**c**) Bax (**d**) Bcl-2 (**e**) IL-1β (**f**) IFN-γ. Data are expressed as mean ± S.E.M. The statistical analysis was performed using one-way ANOVA, followed by Duncan’s multiple range test. ^#^ *p* < 0.05, and ^##^ *p* < 0.01 control/sham vs. malathion. * *p* < 0.05 and ** *p* < 0.01 malathion vs. malathion and Ornipural^®^. ^+^ *p* < 0.05 control/sham vs. malathion and Ornipural^®^. ^&^ *p* < 0.05 control/sham vs. Ornipural^®^.

Figure 3 and Figure 4 showed that the mRNA expression of IL-1β, Bax, and IFN-γ was significantly upregulated in the livers and kidneys of malathion-treated rats, compared to other control groups. In contrast, the expression of Nrf2, Bcl-2, and HO-1 genes was downregulated. Interestingly, intraperitoneal injection of Ornipural^®^ (without malathion) induced a significant upregulation of both Bcl-2 and HO-1 genes, as compared with all other experimental groups. The co-exposure of Ornipural^®^ with malathion induced a marked restoration of deviated genetic expression toward control values.

### 3.5. Changes on 8-Hydroxydeoxyguanosine (8-OHdG) and TNF-Alpha

**Figure 5:**
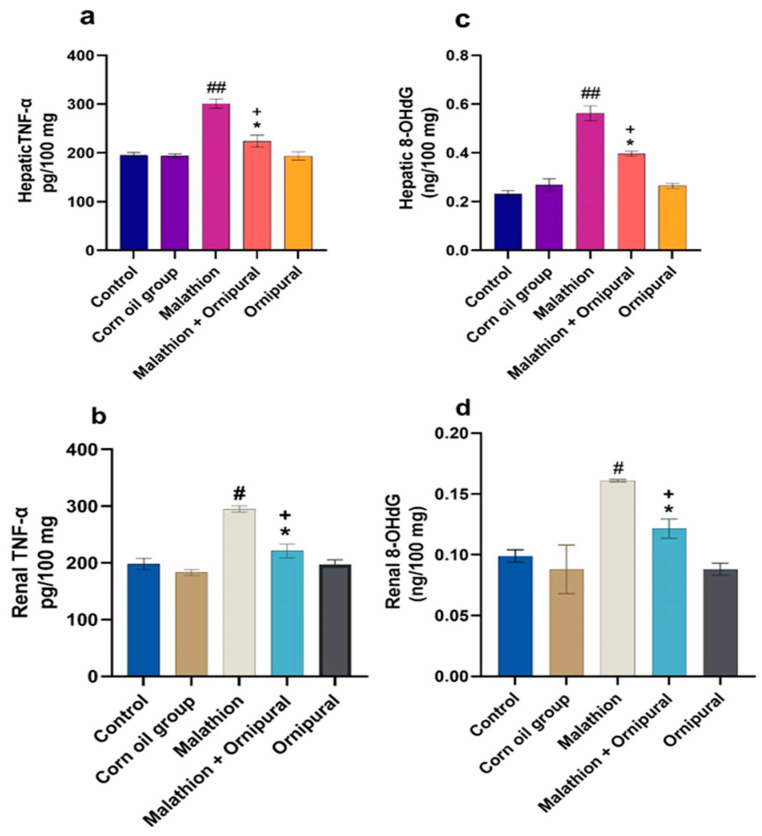
Modulatory effect of Ornipural^®^ against malathion-induced inflammation in hepatic and renal tissue (**a**) hepatic TNF-α (**b**) renal TNF-α (**c**) hepatic 8-OHdG (**d**) renal 8-OHdG. Data are expressed as mean ± S.E.M. The statistical analysis was performed using one-way ANOVA, followed by Duncan’s multiple range test ^#^ *p* < 0.05, and ^##^ *p* < 0.01 control/sham vs. malathion. * *p* < 0.05 malathion vs. malathion and Ornipural^®^. ^+^ *p* < 0.05 control/sham vs. Malathion and Ornipural^®^.

As shown in Figure 5, malathion induced a general state of inflammation, as it increased both hepatic and renal 8-OHdG and TNF-α. Co-administration of Ornipural^®^ with malathion showed significant restoration in the increased 8-OHdG and TNF-α levels.

### 3.6. Histopathological Findings

**Figure 6:**
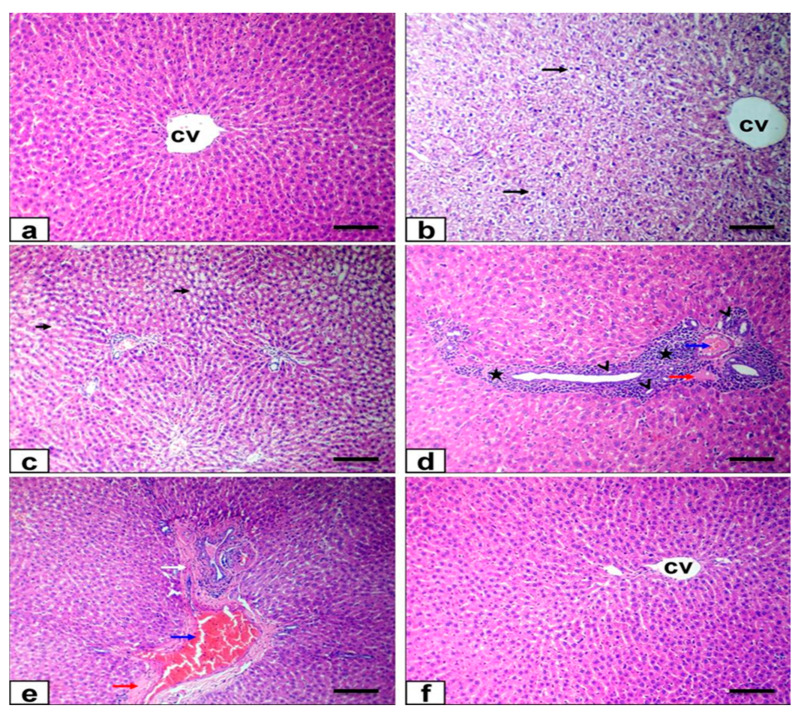
Photomicrograph of liver sections stained with HE: (**a**) liver of control group showing normal histoarchitecture (**b**–**e**) liver of malathion-treated rats showing diffuse hydropic degeneration of hepatocytes (long arrows)and dilatation of hepatic sinusoids (short arrows) beside intense mononuclear cells infiltration (stars), formation of newly formed bile ductules (arrowheads), congestion of portal vein (blue arrow), mild faint eosinophilic albuminous edema (red arrow), and mild fibroplasia (white arrow). (**f**) Ornipural^®^ with malathion-treated rats showing nearly normal histological structure. (Bar = 100 µm), CV = central vein.

Hepatic tissue from the control and Ornipural^®^-treated groups exhibited a normal histological appearance, with intact hepatic architecture in hepatic lobules and common portal areas (Figure 6a). At the same time, the liver sections of malathion-intoxicated rats showed diffuse cytoplasmic vacuolation of the hepatocytes of the hydropic type, characterized by the cytoplasm being replaced by clear fluids and the nucleus not being affected in either shape or location (Figure 6b), particularly at the centrilobular and periportal zones. There was dilatation of hepatic sinusoids, with subsequent atrophy of hepatic cords (Figure 6c). Portal areas also showed moderate to significant thickening due to intense mononuclear cells infiltration, biliary epithelial hyperplasia represented by the formation of newly formed bile ductules, congestion of the portal vein, mild faint eosinophilic albuminous edema, and mild fibroplasia (Figure 6d,e). Co-treatment of Ornipural^®^ with malathion markedly attenuated the harmful effect on cellular morphology. The liver section showed nearly normal histological structure with mild pathological alterations, such as the hepatocytic vacuolation of the hydropic type, as well as the congestion of portal veins and hepatic sinusoids (Figure 6f).

**Figure 7:**
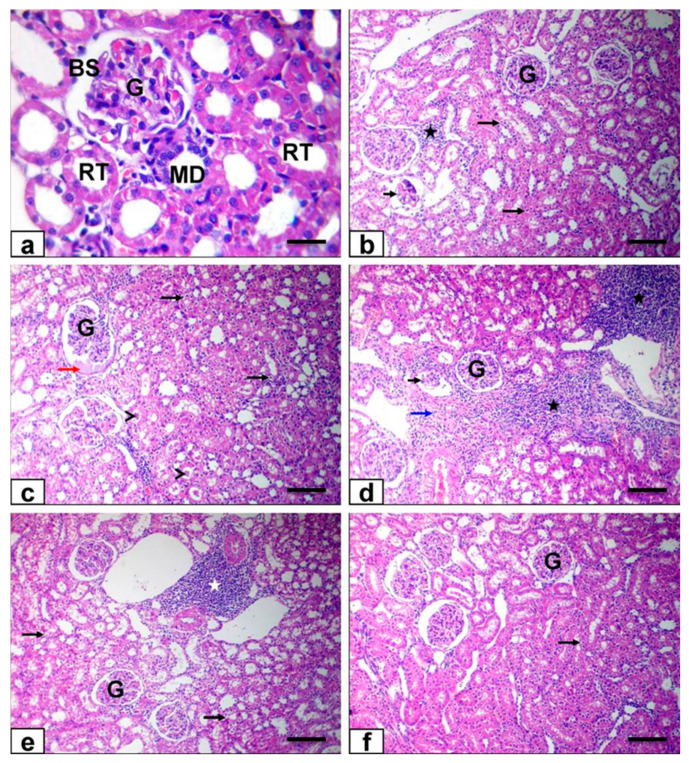
Photomicrograph of kidney sections stained with HE: (**a**) kidney of control group showing normal histoarchitecture (**b**–**e**) kidney of malathion-treated rats showing hydropic vacuolation of the epithelium of renal tubules (long black arrows) and intraluminal hyaline casts (arrowheads), dilatation of Bowman’s space by eosinophilic glomerular filtrate (red arrow), atrophy and necrotic glomerulus (short black arrows), as well as interstitial mononuclear cell infiltrations (white and black stars) and mild fibroplasia (blue arrow) (**f**) Ornipural^®^ with malathion -treated rats showing nearly normal histological structure with mild hydropic epithelial cell degenerations (long black arrows). (Bar of all figures 100 µm, except a 50 µm). G, glomeruli, RT, renal tubules, BS, Bowman’s space, MD, macula densa.

Moreover, the renal tissue from both control and Ornipural^®^-treated rats showed normal morphology of the renal parenchyma, with well-defined glomeruli and renal tubules (Figure 7a). In contrast, kidney sections of malathion-treated rats showed degenerative changes, which were represented by vacuolation of the epithelium of renal tubules and intraluminal hyaline casts (Figure 7b,c). Furthermore, Bowman’s space was dilatated by eosinophilic glomerular filtrate (Figure 7c), leading to atrophy and necrotic glomerulus, as well as vascular congestion of most vascular structures. Interstitial tissue exhibited mononuclear cell infiltrations and mild fibroplasia (Figure 7d,e). On the other hand, hydropic epithelial cell degenerations and vascular congestion were observed at minimal levels in the kidneys of rats that were co-exposed to Ornipural^®^ with malathion (Figure 7f).

## 4. Discussion

The widespread use of the organophosphate insecticide malathion in the veterinary and agricultural fields in many developing countries has resulted in environmental pollution and, subsequently, severe health hazards for both humans and animals [9]. The hepato-renal toxicity of malathion is attributed to oxidative damage, activation of inflammatory cytokines release, disturbance of metabolic functions, promotion of apoptosis, and genes expression modulation [3]. Prolonged malathion exposure is usually associated with the dysfunction of several body organs, especially the liver, kidney, and brain. Similar results were obtained in the present study, as malathion-intoxicated rats suffered from a marked disturbance in the growth performance of biochemical hepatic and renal functions, including liver enzymes, protein and lipid metabolism, urea, and ammonia levels. The observed decrease in growth rate in malathion-poisoned rats may be explained by the insecticide’s diabetic effect, which manifests through the loss of the endocrine function of pancreatic islets (impairment of insulin secretion) and hyperglycemia [37,38]. Moreover, malathion in our study induced an increase in the lipid peroxidation product MDA, DNA oxidation marker 8-OHGdG, and NO indicating an oxidative damage effect. In contrast, the oxidative defense mechanism of hepato-renal tissue was diminished by decreasing the content of GSH, as well as the activity of GPX, SOD, and CAT.

Moreover, the pro-inflammatory cytokine TNF-α shows significant elevation in the hepatic and renal tissues of the malathion-exposed animals, as compared to the control. Furthermore, the expression of IL-1β, Bax, and IFN-γ genes was significantly up-regulated. On the other hand, mRNA expression of Nrf2, Bcl-2, and HO-1 genes was downregulated, indicating activation of inflammatory pathways, apoptosis, fibrosis, and even carcinogenesis. These findings also support the hypothesis mentioned above, which correlates with a malathion-induced inflammatory response in the liver and insulin resistance [39].

Several scientific trials have been performed to ameliorate hepatic and renal damage associated with exposure to malathion [40,41,42]. However, our work is considered the first to investigate using a formula that contains many active ingredients to protect both liver and kidney against malathion toxicity. Moreover, in the present study, we preferred to use commercial malathion products to simulate reality and consider the possibility of contaminants in such products, which may aggravate malathion toxicity [43]. The commercial malathion produces contaminants which seem to be potent inhibitors to carboxylesterases enzymes, which play the principal role in the liver’s malathion and malaoxon detoxification process [44].

Ornipural^®^ is a commercial veterinary product with several active principles (betaine, arginine, ornithine, citrulline, sorbitol, metacresol), and possesses hepato-renal stimulatory and regulatory efficacy. Betaine (trimethylglycine) is a methyl group that is a donor which demonstrates hepatic protection against several causes of liver damage, such as alcohol, hepatitis B virus, experimental cholestasis, and ionizing radiation. The pharmacological effect of betaine is generally related to its anti-inflammatory, anti-apoptotic, and antioxidant properties [45]. The second active ingredient in Ornipural^®^ is arginine, one of the essential amino acids involved in many physiological processes, such as protein deposition, ornithine synthesis, urea removal, immune function enhancement, and improvement of renal function, and is a precursor of nitric oxide synthesis [46]. In fish, arginine shows the potential to stimulate the production of growth-related hormones, such as insulin, glucagon, and growth hormone [47]. The third ingredient in Ornipural^®^ solution is ornithine, which is considered amino acid with improving properties on the urea cycle. The administration of ornithine activates two essential enzymes in the urea cycle, carbamoylphosphate synthetase (CPS) and ornithine transcarbamylase (OTC), that trigger the removal of highly toxic ammonia, which is usually associated with acute liver failure [48]. Citrulline is the fourth active ingredient in Ornipural^®^ solutions. It is considered a natural precursor of arginine. When administered, it become more effective than arginine itself in improving plasma arginine concentration, since citrulline is not destroyed by hepatic or intestinal arginases, and can be converted to arginine directly within the tissues [49]. Sorbitol, for a long time, was used as a diuretic substance. It has recently shown many other beneficial properties, such as decreasing the degradation losses of B12 vitamers (cyanocobalamin, hydroxocobalamin, and methylcobalamin) after exposure to many degradative agents [50].

The co-administration of Ornipural^®^ with malathion in the current study shows significant improvement in growth rate, biochemical biomarkers, oxidative stress-related parameters, gene expression of inflammatory mediators, and histological structures of hepatic and renal tissues when compared with animals that received malathion without Ornipural^®^. The observed beneficial effect of Ornipural^®^ on the growth rate of rats may be attributed to the presence of arginine, which is known to be one of the strongest insulin and growth hormone secretagogues [51,52]. Regarding hepato-renal and metabolic serum biochemical biomarkers, Ornipural^®^ markedly counteracted the adverse impact of malathion via the normalization of AST, ALT, ALP, LDH, ACP, AchE, and paraoxonase in the intoxicated group. The presence of betaine in Ornipural^®^ formula may explain this improvement, since betaine as methyl group doner can reverse the progression of the disruption of liver function, as shown in many previous research articles [53]. Moreover, betaine showed a beneficial role in regulating lipid metabolism during hepatic dysfunction via the promotion of hepatic mitochondrial content and activity [54]. Furthermore, ornithine played the principal role in decreasing serum urea concentration and hyperammonemia, which was detected after malathion exposure, since ornithine acted as a substrate for glutamine synthesis, thereby detoxifying ammonia [55]. In the same context, the combination between arginine and ornithine enhances the detoxification rate of ammonia in cats [56].

As expected, injection of Ornipural^®^ attenuated the oxidative damage induced by malathion in hepatic and renal tissues. This type of protection is attributed to the potent antioxidant activity of betaine [57]. Recently, ornithine also showed a nephroprotective effect against reactive oxygen species (ROS) generation via the activation of Ca^2+^-sensing receptor (CaSR), a G-protein coupled receptor, expressed in the proximal tubule luminal membrane [58].

Concerning the inflammatory response in the liver and kidneys after the co-administration of Ornipural^®^ with malathion, there was a significant decrease in the content of TNF-α in rats treated only with malathion. The obtained data could be explained in light of the anti-inflammatory effect of betaine. Betaine intervention can effectively suppress inflammation [59], and has been shown to reduce the secretion of pro-inflammatory cytokines TNF-α, IL-1β, and IL-6 during fatigue [60]. Results also showed that Ornipural^®^ attenuated the inflammation and apoptosis associated with malathion exposure via the upregulation of the genes expression of Nrf2, Bcl-2, and HO-1, and the downregulation of the genes expression of IL-1β, Bax, and IFN-γ. It was established that Nrf2 represents a crucial regulator of the cellular defense mechanisms against xenobiotic and oxidative stress [61]. The anti-apoptotic effect of Ornipural^®^ was evidenced by the upregulation of anti-apoptotic proteins Bcl-2 and HO-1, and the downregulation of pro-apoptotic protein Bax [62].

Ornipural^®^’s antioxidant and anti-apoptotic activity may be returned to its constituents. Betaine, one of its Ornipural^®^ components, protects the liver of rats against oxidative stress inducers such as thioacetamide and a high-fructose diet [63,64]. The antioxidant action of arginine, another Ornipural^®^ component, and HO-1 overexpression may provide adequate protection against liver injury in combination [65] with the induction of HO-1 without cytotoxic effects as indicated by [66], that HO-1 induction increases without cytotoxicity. The anxiety of this point has brought us to this position. L-arginine induction could be the cause of HO-1.

Arginine is an essential amino acid that helps cells to regenerate, heal wounds, transform proteins, and maintain immunity [67]. Furthermore, arginine supplementation causes an increase in NO generation via iNOS [68], resulting in the creation of HO-1 [69].

Citrulline is another Ornipural^®^ constituent. iNOS can break down arginine into citrulline [70]. Citrulline’s molecular structure is similar to that of arginine; as a result, citrulline can compete with arginine for the active site of iNOS, negatively limiting NO production [71,72]. Following this, increased HO-1 expression was linked to increased Bcl-2 expression [73].

Overall, two enzymes produced reactive oxygen species: (1) in the procedure, aldose reductase used NADPH to convert glucose to sorbitol. In normal physiological settings, sorbitol formation is a muted response [74]. Sorbitol overproduction reduces NADPH availability, which reduces glutathione regeneration and NOS synthase activity, resulting in increased oxidative stress [75]; (2) in the second step, sorbitol dehydrogenase oxidizes sorbitol to fructose, while producing NADH. NADH oxidases may use increased NADH to increase superoxide production [75]. Increased sorbitol pathway activity causes oxidative stress in diabetic complication tissue sites [76]. SDH is a polyol pathway enzyme that converts sorbitol to fructose in the presence of NAD. The activity of SDH is increased in diabetics, resulting in more fructose available for glycosylation than glucose [1]. To reduce the oxidative stress cascade effect in diabetic rats, the availability of sorbitol should be reduced [77]. Sorbitol accumulation causes diabetic complications [78]. The beneficial effects of sorbitol were attributed to its ability to restore redox status and reduce markers of apoptosis, inflammation, and catabolism involved in cartilage damage [79]. This is supported by [80], who reported the antioxidant activity of sorbitol.

Sorbitol had hypoglycemic effects in normal and type 2 diabetic rats by increasing muscle glucose uptake and decreasing intestinal glucose absorption. Sorbitol may therefore be studied as an anti-hyperglycemic sweetener [81]. Sorbitol is widely used due to its many health benefits [82]. According to a previous study, sorbitol may be helpful for glycemic control in both normoglycemic and diabetic subjects [83]. Sorbitol therapy improved psychomotor performance [84]. Gut bacteria metabolize sorbitol, sorbitol therapy improved psychomotor performance in cirrhotic patients, and previous studies that used sorbitol as a control underestimated the benefits of lactulose [84]. The primary health concern associated with sorbitol consumption is gastrointestinal disturbances, as sorbitol is not completely digested in the small intestine [85].

The previously discussed methods of Ornipural^®^ protection against malathion-induced hepato-renal damage were supported by histopathological examination, since most pathologic microscopic alterations associated with malathion exposure were attenuated under Ornipural^®^ treatment.

## 5. Conclusions

Ornipural^®^ as a therapeutic solution containing several active ingredients showed potent protection against malathion’s biochemical, oxidative, and inflammatory effects, probably via the restoration of biochemical parameters, enhancement of the antioxidant defense mechanisms, and reduction in the generation of inflammatory mediators.

## Figures and Tables

**Table 1:** Primers for gene expression by RT-PCR.

Gene	Direction	Primer Sequence	Accession Number
Bax	Sense	GGCGAATTGGCGATGAACTG	NM_017059.2
	Antisense	ATGGTTCTGATCAGCTCGGG	
Bcl-2	Sense	GATTGTGGCCTTCTTTGAGT	NM_016993.1
	Antisense	ATAGTTCCACAAAGGCATCC	
GAPDH	Sense	TCAAGAAGGTGGTGAAGCAG	NM_017008.4
	Antisense	AGGTGGAAGAATGGGAGTTG	
IL-1β	Sense	ACC CAA GCA CCT TCT TTT CCT T	NM_031512.2
	Antisense	ACG GGA AAC CCA TCA CCA T	
HMOX1	Sense	AGCATGTCCCAGGATTTGTC	NM_012580.2
	Antisense	TCACCAGCTTAAAGCCTTCC	
NRF2	Sense	TTGTAGATGACCATGAGTCGC	NM_031789
	Antisense	TGTCCTGCTGTATGCTGCTT	
IFN-γ	Sense	AGGTGAACAACCCACAGAT	NM_138880.3
	Antisense	CTTCTTATTGGCACACTCTCTAC	

*Bax*, Bcl-2-associated X protein. *Bcl-2*, B-cell lymphoma 2. IL-1β, interleukin 1b. *HMOX1*, Haemoxygenase-1.IFN-γ, Interferon-gamma. GAPDH, glyceraldehyde-3-phosphate dehydrogenase. Nrf2, The nuclear factor erythroid 2-related factor 2.

**Table 2:** Modulatory effect of Ornipural^®^ against the impact of malathion on growth performance.

	Control	Corn Oil Group (Sham)	Malathion	Malathion + Ornipural^®^	Ornipural^®^
Initial body Weight (g)	135.05 ± 5.14	137.5 ± 5.3	144.45 ± 5.4	139.48 ± 6.48	141.45 ± 4.15
Final body Weight (g)	268.15 ± 6.45	267.9 ± 6.5	231.01 ± 6.9 ^##^	248.15 ± 8.45 *^+^	265.1 ± 5.3
Bodyweight gain	133.15 ± 4.8	130.4 ± 7.45	86.56 ± 4.5 ^##^	108.67 ± 4.15 *^+^	123.65 ± 4.15
Absolute Weight of Liver (g)	6.45 ± 0.52	6.63 ± 0.15	5.321 ± 0.22	5.48 ± 0.6	6.51 ± 0.2
Relative liver Weight (g/100 g BW)	2.40 ± 0.14	2.47 ± 0.15	2.30 ± 0.14	2.20 ± 0.14	2.45 ± 0.01
Absolute Weight of Kidney (g)	1.79 ± 0.12	1.77 ± 0.14	1.69 ± 0.014	1.75 ± 0.1	1.76 ± 0.05
Relative Weight of Kidney (g/100 g BW)	0.66 ± 0.01	0.660 ± 0.014	0.733 ± 0.01	0.70 ± 0.01	0.66 ± 0.1

Data are expressed as mean ± S.E.M. The statistical analysis was performed using one-way ANOVA, followed by Duncan multiple range test. ^##^ *p* < 0.01, control/sham vs. malathion. * *p* < 0.05 malathion vs. malathion and Ornipural^®^. ^+^ *p* < 0.05 control/sham vs. malathion and Ornipural^®^.

**Table 3:** Modulatory effect of Ornipural^®^ against the harmful impact of malathion on hepato-renal functional biomarkers in the serum.

	Control	Corn Oil Group	Malathion	Malathion + Ornipural^®^	Ornipural^®^
AST (U/mL)	80.58 ± 5.2	79.00 ± 5.9	172.86 ± 8.6 ^##^	89.82 ± 4.5 *^+^	75.26 ± 2.3
ALT (U/mL)	35.15 ± 3.1	34.46 ± 2.5	77.07 ± 4.2 ^##^	48.43 ± 3.2 *^+^	36.29 ± 1.5
ALP (U/L)	85.20 ± 4.45	83.53 ± 3.6	202.49 ± 15.14 ^##^	99.82 ± 5.3 **^+^	77.88 ± 4.6 ^&^
LDH (U/L)	194.66 ± 10.2	195.75 ± 12.3	431.95 ± 17.2 ^##^	298.03 ± 10.2 **^+^	192.87 ± 9.01
ACP(U/L)	101.12 ± 11.2	102.14 ± 10.2	181.3 ± 10.14 ^##^	119.34 ± 10.45 *^+^	100.14 ± 2.9
Bilirubin (mg/dL)	5.22 ± 0.14	5.10 ± 0.10	7.15 ± 0.6 ^#^	6.12 ± 0.4 *^+^	5.01 ± 0.4
Total protein (g/L)	5.10 ± 0.5	5.00 ± 0.1	3.42 ± 0.4 ^#^	4.20 ± 0.2 *^+^	4.95 ± 0.5
Albumin (g/L)	4.02 ± 0.4	3.94 ± 0.6	2.92 ± 0.1 ^#^	3.47 ± 0.1 *^+^	3.91 ± 0.1
Triglycerides (g/L)	110.29 ± 6.3	108.12 ± 10.4	67.78 ± 1.3 ^##^	84.23 ± 4.5 *^+^	105.05 ± 3.8
HDL-C (mg/dL)	65.84 ± 4.45	63.01 ± 6.48	49.12 ± 3.8 ^##^	53.14 ± 4.7 *^+^	66.8 ± 5.6
LDL-C (mg/dL)	101.14 ± 6.1	100.4 ± 6.2	125.14 ± 6.4 ^##^	114.12 ± 4.5 *^+^	99.15 ± 5.2
Cholesterol (mg/dL)	77.44 ± 4.6	79.88 ± 3.4	149.10 ± 9.5 ^##^	104.20 ± 5.6 *^+^	71.19 ± 3.5 ^&^
Uric acid (mg/dL)	25.46 ± 3.6	24.96 ± 1.01	80.05 ± 7.5 ^##^	41.05 ± 2.5 *^+^	20.97 ± 1.01
Urea (mg/dL)	21.83 ± 2.3	21.40 ± 1.0	73.99 ± 4.5 ^##^	40.26 ± 3.2 *^+^	18.35 ± 1.04
Creatinine (mg %)	0.66 ± 0.01	0.64 ± 0.01	2.05 ± 0.3 ^#^	1.76 ± 0.1 *^+^	0.57 ± 0.1
AChE (U/L)	250.01 ± 10.15	245.15 ± 11.2	75.96 ± 5.8 ^##^	126.15 ± 6.48 *^+^	233.15 ± 12.4
Paraoxonase (U/L)	176.14 ± 13.1	177.5 ± 12.4	120.14 ± 5.4 ^##^	138.56 ± 7.14 *^+^	181.14 ± 13.1
Ammonia (μmol/L)	128.15 ± 10.12	131.14 ± 9.48	256.1 ± 17.69 ^##^	186.14 ± 12.14 **^+^	130.14 ± 12.69

Data are expressed as mean ± S.E.M. The statistical analysis was performed using one-way ANOVA, followed by Duncan’s multiple range test. ^#^ *p* < 0.05, and ^##^ *p* < 0.01. control/sham vs. malathion. * *p* < 0.05 and ** *p* < 0.01 malathion vs. malathion and Ornipural^®^. ^+^ *p* < 0.05 control/sham vs. malathion and Ornipural^®^. ^&^ *p* < 0.05 control/sham vs. Ornipural^®^.

## Data Availability

The data presented in this study are available on request from the corresponding author.
